# AMPH1 functions as a tumour suppressor in ovarian cancer via the inactivation of PI3K/AKT pathway

**DOI:** 10.1111/jcmm.15400

**Published:** 2020-05-31

**Authors:** Yajun Chen, Wenjiao Cao, Lihua Wang, Tianying Zhong

**Affiliations:** ^1^ Department of Clinical Laboratory Nanjing Maternity and Child Health Care Hospital Women’s Hospital of Nanjing Medical University Nanjing China; ^2^ The international Peace Maternity and Child Health Hospital School of Medicine The China Welfare Institute Shanghai Jiao Tong University Shanghai China; ^3^ Shanghai Key Laboratory of Embryo Original Diseases Shanghai China; ^4^ Shanghai Municipal Key Clinical Specialty Shanghai China

**Keywords:** AKT pathway, AMPH1, anti‐oncogene, ovarian cancer, PI3K, tumour suppressor

## Abstract

AMPH1, an abundant protein in nerve terminals, plays a critical role in the recruitment of dynamin to sites of clathrin‐mediated endocytosis. Recently, it is reported to be involved in breast cancer and lung cancer. However, the impact of AMPH1 on ovarian cancer is unclear. In this study, we used gain‐of‐function and loss‐of‐function methods to explore the role of AMPH1 in ovarian cancer cells. AMPH1 inhibited ovarian cancer cell growth and cell migration, and promoted caspase‐3 activity, resulting in the increase of cell apoptosis. In xenograft mice model, AMPH1 prevented tumour progression. The anti‐oncogene effects of AMPH1 on ovarian cancer might be partially due to the inhibition of PI3K/AKT signalling pathway after overexpression of AMPH1. Immunohistochemistry analysis showed that the staining of AMPH1 was remarkably reduced in ovarian cancer tissues compared with normal ovarian tissues. In conclusion, our study identifies AMPH1 as a tumour suppressor in ovarian cancer in vitro and in vivo. This is the first evidence that AMPH1 inhibited cell growth and migration, and induced apoptosis via the inactivation of PI3K/AKT signalling pathway on ovarian cancer, which may be used as an effective strategy.

## 1 INTRODUCTION

Amphiphysin 1 (AMPH1), a phosphoprotein expressed at high levels in neurons, participates in synaptic vesicle endocytosis and neurite outgrowth.^1^ It belongs to a protein family conserved from yeast to humans, playing pleiotropic roles in actin function, and regulation of growth control.^1^ AMPH1 interacts with other endocytic proteins, including dynamin and adaptor Protein Complex 2, involved in the recruitment of dynamin to sites of chathrin‐mediated endocytosis.^2^, ^3^ The recruitment mediates the last step in synaptic vesicle endocytosis, an essential neurophysiologic component in synaptic transmission.^4^ AMPH1 also serves as a multifunctional adaptor connecting clathrin coat proteins, dynamin, synaptojanin and lipid membranes.^2^ In the proline‐rich domain, AMPH1 includes several phosphorylation sites for protein kinases, like cyclin‐dependent kinase 5, a member of the cyclin‐dependent protein kinase family, which plays a role in neuronal migration and neurite outgrowth via its action on the actin cytoskeleton.^1^, ^2^

AMPH can express in non‐neuronal tissues. The 108 kD isoform of AMPH1 is the predominant isoform that expressed outside the brain in human, and it represents an alternatively spliced variant of neuronal AMPH1 missing a 42 amino acid insert.^5^ AMPH1 is first identified as a human autoantigen in neurological paraneoplastic autoimmune diseases with breast cancer,^6^ and then AMPH1 antibodies are detected in the serum of patients with small‐cell lung cancer.^7^ Further study reported a link between AMPH1 expression in cancer and AMPH1 autoimmunity, and this is the first proof suggesting AMPH family members may be associated with cancer.^5^ Subsequently, Otsuka et al^8^ demonstrated that AMPH1 inhibited the adhesion and spreading of HeLa cells as well as migration of melanoma cells by binding to vitronectin. In recent studies, the association between AMPH1 and tumour progression is well investigated. It is reported that AMPH1 expressed lower in SKMES‐1 and A549 cells.^9^ In addition, AMPH1 can function as a tumour suppressor in breast cancer cells. Chen et al^10^ found that inhibition of AMPH1 induced cell proliferation, cell migration and cell cycle progression, but inhibited cell apoptosis, which might be partially due to the activation of EMT and ERK pathways. In lung cancer, Yang et al^11^ also identified the anti‐oncogenic function of AMPH1 in vitro and in vivo, and the suppression effects were mediated by Ras‐Raf‐MEK‐ERK signalling pathway. However, up to now, no study reports the influences of AMPH1 on ovarian cancer.

Ovarian cancer, the seventh most commonly diagnosed carcinoma in the world, is one of the most aggressive type's gynaecologic cancers.^12^, ^13^ Approximately 85% ovarian cancer cases are diagnosed at an advanced stage (III/IV) due to the lack of screening for early diagnosis of ovarian cancer.^12^ As an aggressive tumour, ovarian cancer is characterized by the tendency of metastasizing early.^14^ Recent years effective treatment strategies have developed diversely; however, survival outcomes barely improved.^15^ Therefore, it is imperative to identify novel therapeutic strategy for ovarian cancer.

Here, the roles of AMPH1 in ovarian cancer were systematically investigated, and the mechanism underlying these effects was also explored. This study demonstrated that AMPH1 functioned as a tumour suppressor in ovarian cancer. Specifically, the anti‐oncogene effect of AMPH1 may through induce apoptosis via promoting caspase‐3 activity and the inactivation of PI3K/AKT signalling pathway.

## 2 MATERIALS AND METHODS

### 2.1 Tissue samples from patients with ovarian cancer

A total 15 ovarian cancer specimens and 15 normal ovarian tissues were obtained from Nanjing Maternity and Child Health Care Hospital. All patients have written informed consent. Fresh specimens were immediately fixed in formalin and embedded in wax for immunohistochemistry.

### 2.2 Cell culture

Two human ovarian cancer cell lines, Caov‐3 and Skov3 cells, were obtained from American Type Culture Collection (ATCC, USA). Cells were cultured in RPMI 1640 with 10% foetal bovine serum (FBS, Gibco) at 37°C in a humidified incubator filled with 5% CO_2_.

### 2.3 Cell transfection

The stable cell lines with AMPH1 knockdown were generated by integration of lentiviral shRNA vectors specific for AMPH‐1. The shRNA against AMPH‐1 shown as follows: 5′‐GCGAGAACUCCGAGGAUAUTT‐3′. The PCMV‐AMPH overexpression plasmid (abnova) was used to construct AMPH1 overexpression cell lines.

### 2.4 Western blot assay

Total proteins extracted from all cells were resolved on SDS‐PAGE. And then, proteins on gel were transferred to PVDF membranes which were blocked with 5% BSA in TBST for 1 hour and incubated with corresponding primary antibodies overnight at 4°C. AMPH‐1, p‐PI3K(Tyr458), P‐AKT(Ser473), PI3K, AKT and β‐actin antibodies were purchased from Proteintech and Cell Signaling. The membranes were incubated with secondary antibody for 1 hour at room temperature. Finally, an enhanced chemiluminescence system was further performed to detect protein levels.

### 2.5 QRT‐PCR

Total RNA was extracted by using TRIzol reagent (TaKaRa Bio). And then, RNA was reverse‐transcribed into complementary DNA (cDNA) by using a First‐Strand cDNA Synthesis kit (TaKaRa Bio). SYBR mixed with cDNA was used. The relative expression level of AMPH1 in each type of cell was analysed by using 2−ΔΔCt method. Primers were listed below.

### 2.6 The caspase‐3 activity assay

The Caspase‐3 Activity Assay was done according to the protocol of the Caspase‐3 Activity Assay Kit (Cell Signaling, #5723). It contains a fluorogenic substrate (N‐Acetyl‐Asp‐Glu‐Val‐Asp‐7‐amino‐4‐methylcoumarin or Ac‐DEVD‐AMC) for caspase‐3. During the assay, activated caspase‐3 cleaves this substrate between DEVD and AMC, generating highly fluorescent AMC that can be detected using a fluorescence reader with excitation at 380 nm and emission between 420 and 460 nm.

### 2.7 Cell growth assay

Cell growth was detected via Cell Counting Kit‐8 (CCK‐8) assay. Cells were transformed to 96‐well plates at a density of 3000 cells/well. CCK‐8 experiment was performed according to the manufacturer's instructions. Absorbance at 490 nm was obtained by a microplate reader (BioTek Instruments, Inc). OD values were determined after 24, 48, 72 and 96 hours.

### 2.8 Cell apoptosis assay

Annexin‐VFITC Apoptosis Detection Kit I (BD Biosciences) was used for analysing apoptosis. cells were resuspended by cold PBS at a density of 1106 cells/ml, and incubated with PI and Annexin‐VFITC staining, followed by incubation at room temperature for 15 minutes. Flow cytometry was used to analyse apoptosis.

### 2.9 Wound healing assay

Cells were transformed to six‐well plates with 10% FBS. When 80% of the well was covered with cells, a 100 μl pipette tip was used to scrap the cells. As a result, wounds were generated. Then, cells were incubated at 37°C. After 24 hours, the migration distance of cells was determined. The width ratio was calculated by the wound width/the distance measured at 0 hour.

### 2.10 Immunohistochemistry

Human tumour tissues embedded in paraffin were sliced into 5 μm sections for staining. To retrieve antigenic activity, the deparaffinized and rehydrated sections were heated in citrate buffer at 121°C for 30 minutes. Blocking was done by incubation with 10% goat serum at room temperature for 30 minutes. Sections were incubated with AMPH1 antibody for overnight at 4°C, followed by staining with secondary antibody for 1 hour at room temperature. Sections were finally stained with 3, 3‐diaminobenzidine tetrahydrochloride and counterstained with haematoxylin.

AMPH1 staining was evaluated by pathologists who were blinded to the sample origins and the patient outcomes. The widely accepted German semi‐quantitative scoring system was used to score staining intensity and extent in different areas. Each specimen was scored according to the intensity of the nucleic, cytoplasmic and membrane staining (weak staining = 1, moderate staining = 2 and strong staining = 3) and the proportion of stained cells (0%‐5% = 0, 5%‐25% = 1, 26%‐50% = 2, 51%‐75% = 3 and 76%‐100% = 4). The final immunoreactivity score was determined by multiplying the intensity score by the proportion score. All IHC scores were ranged from 0 (the minimum score) to 12 (the maximum score).

### 2.11 Xenograft mice model

Female BALB/c nude mice aged 4‐ to 6‐week old were used. Mice were divided to three groups, and subcutaneously injected with ShAMPH1 or AMPH‐OE Skov3 cells, respectively. After 21 days, mice were sacrificed. Tumour volume and weight in each group were measured.

### 2.12 Statistical analysis

Data are presented as mean ± SEM. All experiments were performed at least three times. Data were assessed using the software SPSS 19.0. *P* < .05 was regarded statistically significant difference.

## 3 RESULTS

### 3.1 AMPH1 significantly suppressed the cell proliferation of ovarian cancer cell lines

**Figure 1:**
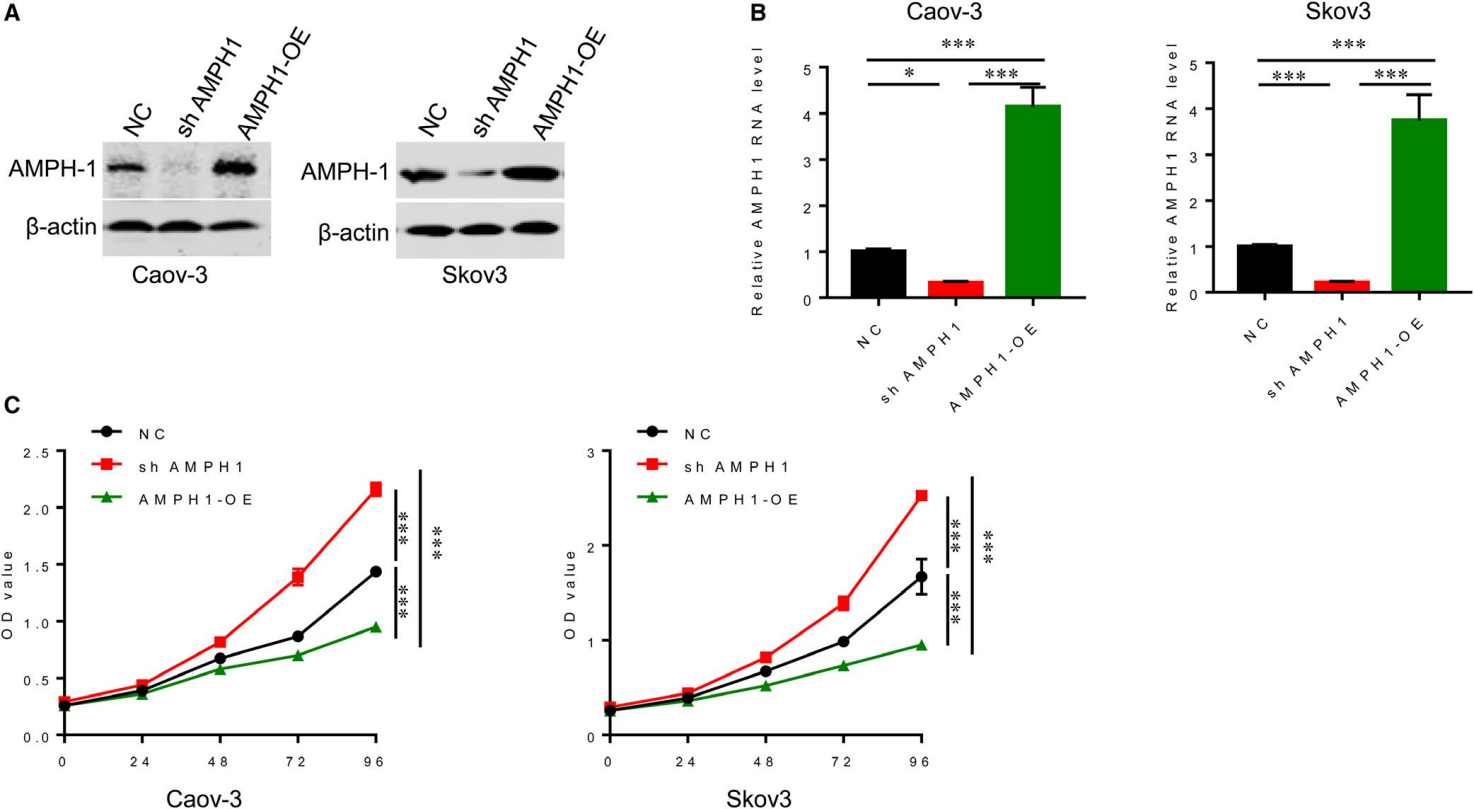
AMPH1 significantly suppressed the cell proliferation of ovarian cancer cell lines. A, Western blot assay showed that the protein level of AMPH1 in AMPH‐1 silenced (ShAMPH1) or overexpressed (AMPH‐OE) Caov‐3 and Skov3 cells. B, qRT‐PCR showed the mRNA level of AMPH1 in ShAMPH1 or AMPH‐OE Caov‐3 and Skov3 cells. C, MTT assay showed that knockdown of AMPH1 significantly promoted cell growth, and overexpression of AMPH1 suppressed the cell proliferation of ovarian cancer cell lines. * *P* < .05. ***P < .001

To detect the roles of AMPH1 in ovarian cancer, gain‐of‐function and loss‐of‐function methods were used. We generated the AMPH‐1 silenced (ShAMPH1) or overexpressed (AMPH‐OE) stable Caov‐3 and Skov3 cells. Western blotting and qRT‐PCR were performed to verify the protein and mRNA levels of AMPH1 respectively after transfection. As shown in Figure 1A,B, AMPH1 protein and mRNA levels were decreased in sh AMPH1 cells. Conversely, the protein and mRNA levels increased in AMPH‐OE cells (Figure 1A,B). Subsequently, we further explored the ability of cell proliferation. MTT results showed that knockdown of AMPH1 remarkably aggravated the accumulation of cells compared with cells without transfection (Figure 1C). However, overexpression of AMPH1 significantly inhibited the growth of Caov‐3 and Skov3 cells (Figure 1C).

### 3.2 AMPH1 significantly induced the cell apoptosis of ovarian cell lines

**Figure 2:**
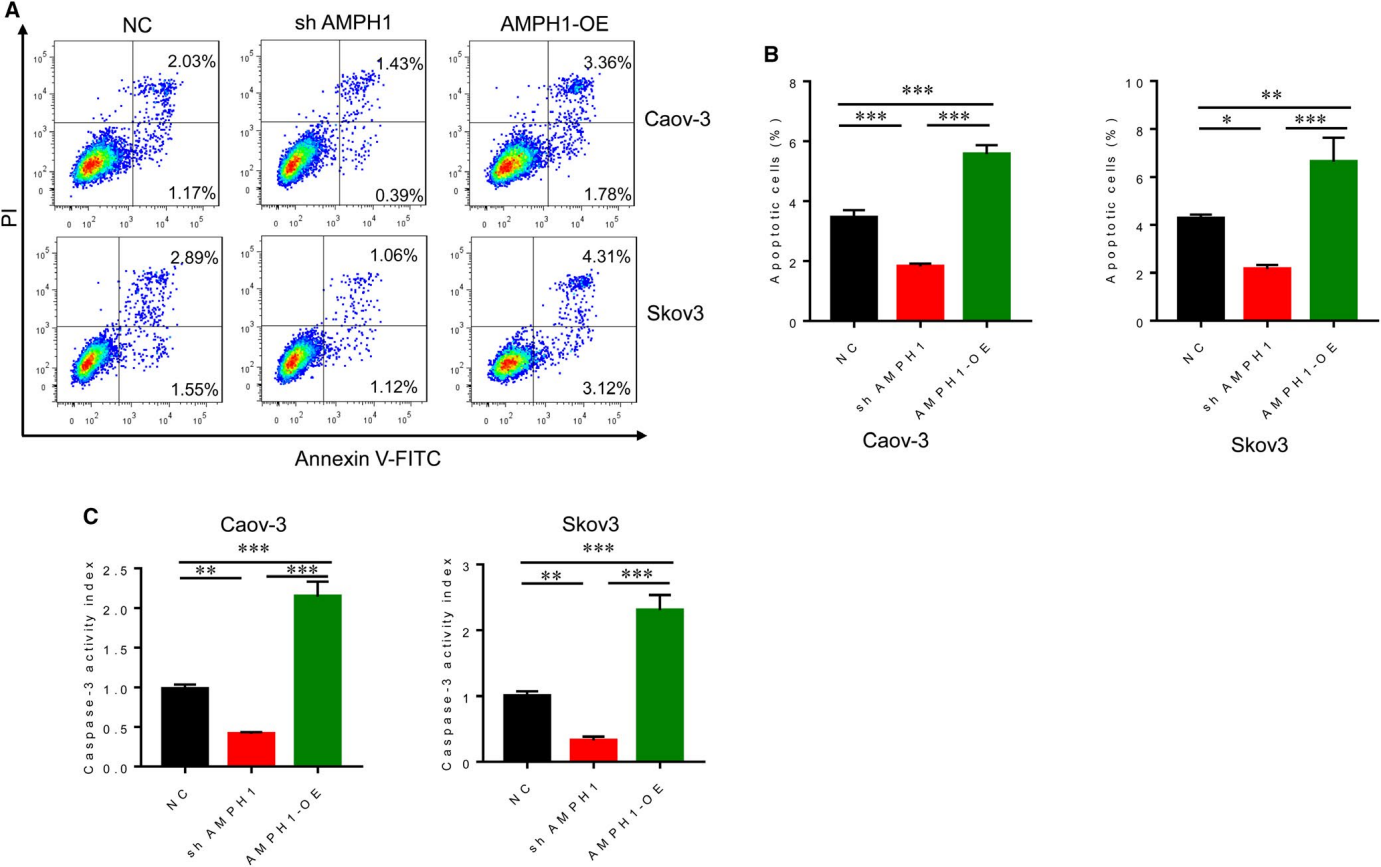
AMPH1 induced cell apoptosis and promoted caspase‐3 activity. A, Flow cytometry results showed that knockdown of AMPH1 suppressed ovarian cancer cell apoptosis, whereas overexpression of AMPH1 stimulates cell apoptosis. B, The percentage of apoptotic cells in each type of cell was calculated. C, Knockdown of AMPH1 inhibited caspase‐3 activity, and overexpression of AMPH1 promoted caspase‐3 activity. *P < .05. **P < .01. ***P < .001

**Figure 3:**
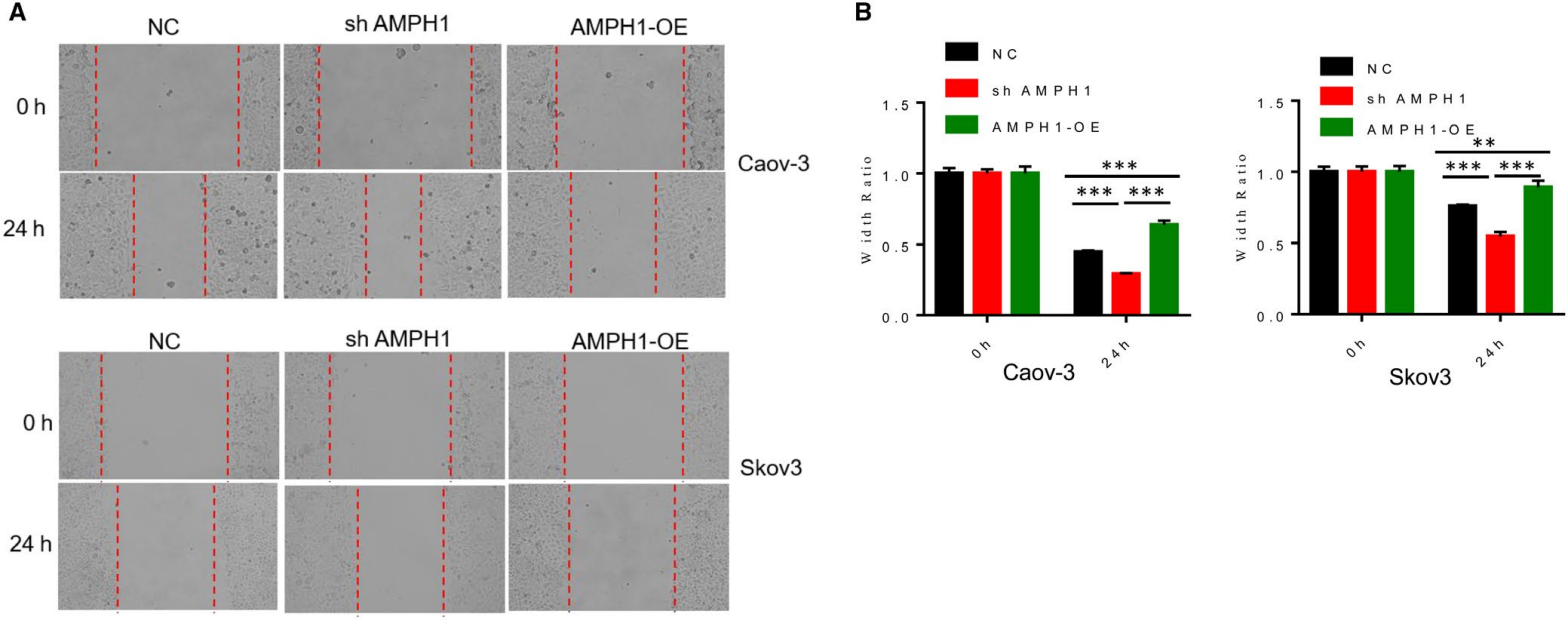
AMPH1 significantly inhibited cell migration of ovarian cancer cell lines. A, Wounding healing assay showed that AMPH1 suppressed cell migration in Caov‐3 and Skov3 cells. Migration of cells to the wound was visualized at 0 and 24 h with a microscope. B, The width ratio was calculated by the wound width/the distance measured. **P < .01. ***P < .001

We next evaluated the effect of AMPH1 on apoptosis in Caov‐3 and Skov3 cells. Flow cytometry results showed that the absence of AMPH1 significantly prevented cell apoptosis, whereas the increase of AMPH1 expression in ovarian cancer cell lines significantly induced cell apoptosis (Figure 2A,B). One of the best‐known markers of apoptosis is the proteolytic cleavage of pro‐caspase‐3 into its active form.^16^, ^17^ Caspase‐3, a main executor of apoptosis, specifically cleaved substrates and finally results in DNA fragmentation and cell apoptosis. To further define the apoptosis involved by AMPH1, we detected the activity of caspase‐3 in Caov‐3 and Skov3 cells. AMPH1 knockdown markedly suppressed caspase‐3 activity, and AMPH1 overexpression markedly promoted caspase‐3 activity (Figure 3C), indicating AMPH1 was as a tumour suppressor to promote ovarian cancer cell apoptosis.

### 3.3 AMPH1 significantly inhibited cell migration of ovarian cancer cell lines

Ovarian cancer is one of the most aggressive type's gynaecologic cancers in the world characterized by the tendency of metastasizing early.^13^, ^14^ Metastasis is generally connected with poor prognosis. Thus, here, we evaluated the impact of AMPH1 on the cell migration in Caov‐3 and Skov3 cells by using wounding healing assay. Migration of cells to the wound was visualized at 0 and 24 hours with a microscope (Figure 3A). The width ratio was calculated by the wound width/the distance measured at 0 hour (Figure 3B). The results showed that the width ratio was higher in ovarian cancer cells with sh AMPH1 compared with control cells, and lower in cells with AMPH1 overexpression. These findings suggest that silence of AMPH1 induced cell migration; in turn, the accumulation of AMPH1 inhibited the ability of cell migration.

### 3.4 AMPH1 inhibited the activation of PI3K/AKT signalling pathway

**Figure 4:**
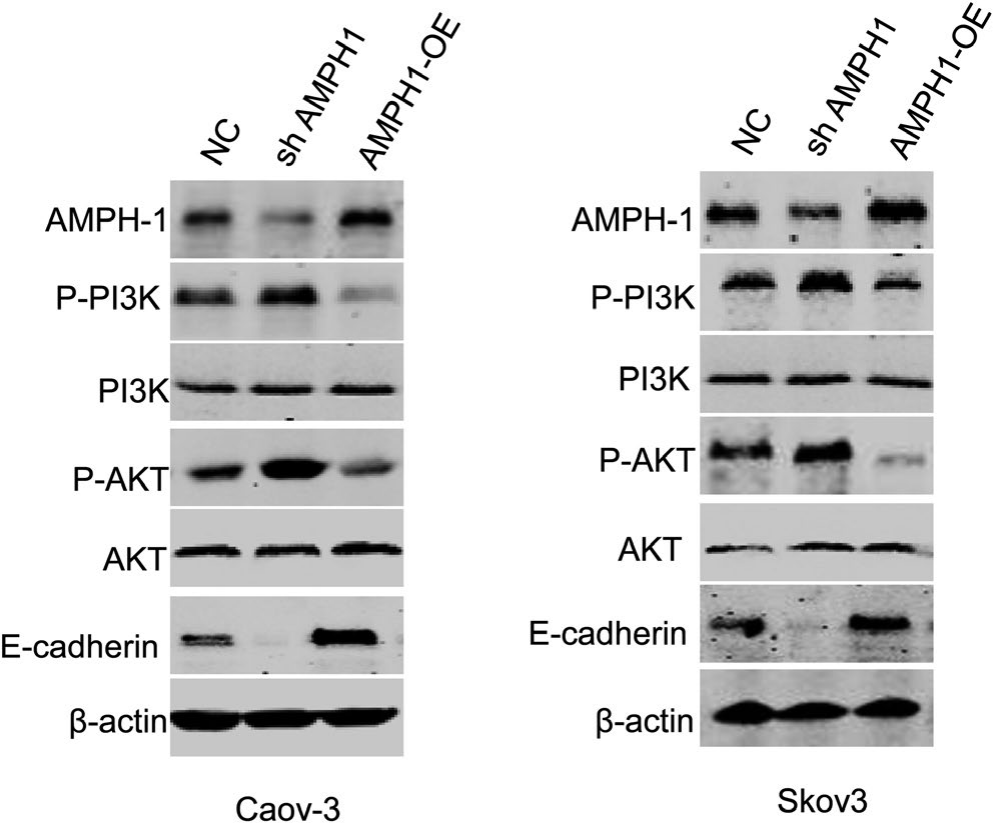
AMPH1 inhibited the activation of PI3K/AKT signalling pathway in ovarian cancer cells. Western blot assay showed that knockdown of AMPH1 increased the protein levels of p‐PI3K and p‐AKT, whereas overexpression of AMPH1 inhibited these proteins levels. In addition, silencing AMPH1 inhibited the expression of E‐cadherin

The PI3K/AKT signalling pathway is one of many mechanisms that regulate cell cycle and cell apoptosis, and dysregulation of a component in this pathway leads to cancer.^18^ Therefore, we further detected the association between AMPH1 and PI3K/AKT signalling pathway. Inhibiting AMPH1 expression induced the accumulation of p‐PI3K and p‐AKT proteins, and AMPH1 overexpression suppressed these proteins levels, revealing AMPH1 might regulate ovarian cancer progression via PI3K/AKT signalling pathway (Figure 4). Loss of E‐cadherin gene expression causes dysfunction of cell junction system, allowing cancer cell invasion and metastasis. To elucidate the underlying mechanism by which AMPH1 regulates cell invasion, we investigated expression of E‐cadherin in Caov‐3 and Skov3 cells. Compared with shN cells, silencing AMPH1 had a decreased expression of E‐cadherin at protein levels (Figure 4), suggesting AMPH1 regulated the E‐cadherin.

### 3.5 AMPH1 significantly inhibited ovarian tumour progression in vivo

**Figure 5:**
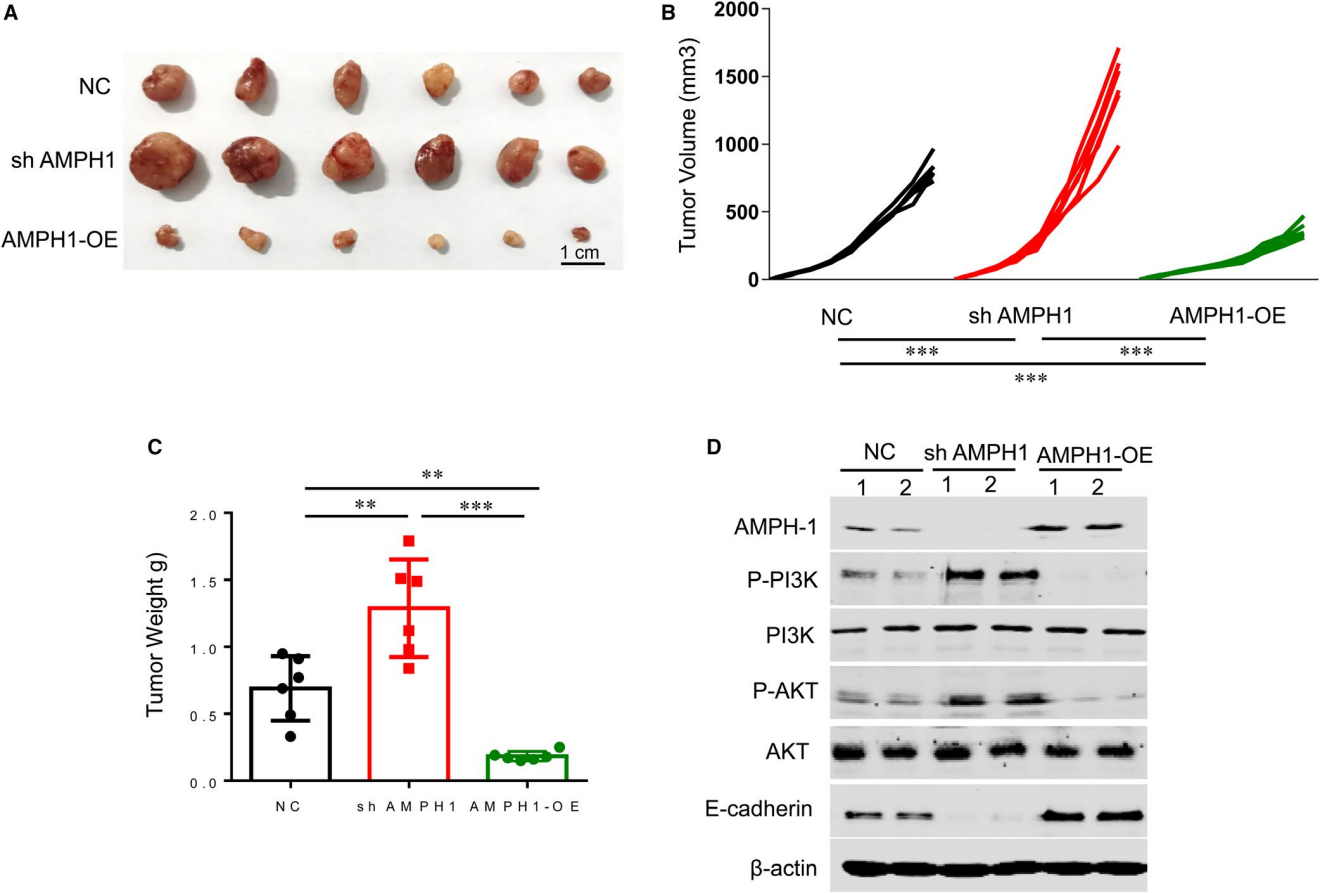
AMPH1 inhibited tumour growth in vivo. A, The images of tumours injection with ShAMPH1 or AMPH‐OE Skov3 cells. B, Knockdown of AMPH1 increased tumour volume, whereas overexpression of AMPH1 decreased tumour volume. C, Knockdown of AMPH1 increased tumour weight, whereas overexpression of AMPH1 decreased tumour weight. **P < .01. ***P < .001. D, The protein expression of AMPH1, p‐PI3K, p‐AKT and E‐cadherin in xenograph tumours

Three types of Skova3 cells (control, sh AMPH1, AMPH‐OE) were inoculated in mice. After 21 days, mice were sacrificed. Tumour volume and weight in each group were measured. As shown in Figure 5, the absence of AMPH1 increased tumour volume and tumour weight, whereas overexpression of AMPH1 decreased tumour volume and tumour weight (Figure 5A‐C). These results suggest that AMPH1 inhibited ovarian tumour progression in xenograft mouse model. Furthermore, we detected the AMPH1, E‐cadherin and PI3K/AKT levels in tumours derived from cells silenced and high expressed AMPH1 and found the expression of AMPH1 still decreased in the dissected tumours (Figure 5D).

### 3.6 The staining of AMPH1 is decreased in human ovarian cancer tissues compared with normal ovarian tissues

**Figure 6:**
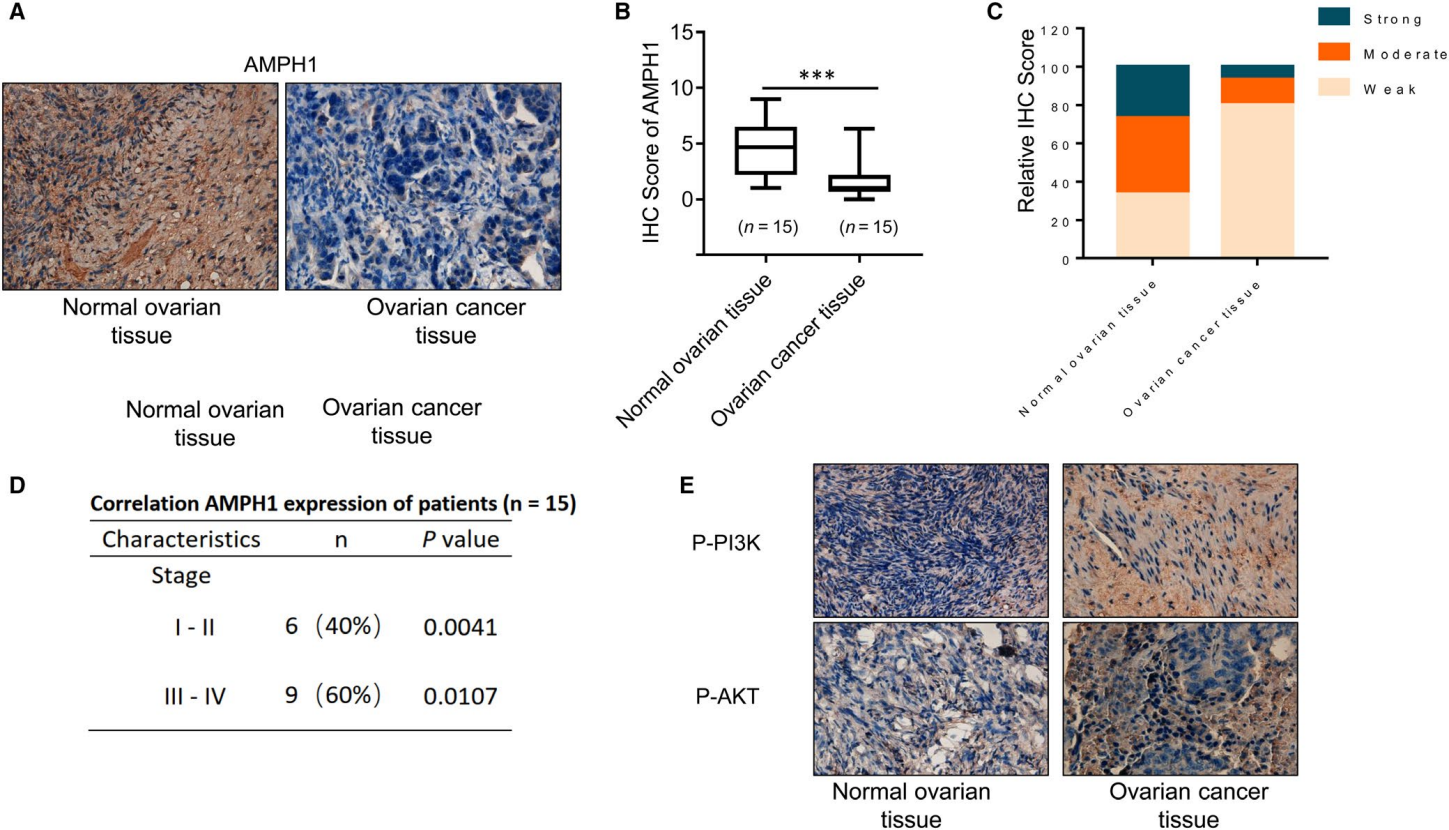
The staining of AMPH1 was remarkably reduced in human ovarian cancer samples compared with normal ovarian tissues. A, Immunohistochemistry analysis of AMPH1 in human ovarian cancer samples and normal ovarian tissues. B, Each specimen was scored according to the proportion of stained cells (0%‐5% = 0, 5%‐25% = 1, 26%‐50% = 2, 51%‐75% = 3 and 76%‐100% = 4). C, Each specimen was scored according to the intensity of the nucleic, cytoplasmic and membrane staining (weak staining = 1, moderate staining = 2 and strong staining = 3). IHC score showed that the staining of AMPH1 was remarkably reduced in ovarian cancer tissues compared with normal ovarian tissues. ***P < .001. D, The clinicopathologic characteristics of patients and the correlation of AMPH1 were shown. *P* value was determined by Student's *t* test. E, Immunohistochemistry analysis of p‐PI3K and p‐AKT in human ovarian cancer samples and normal ovarian tissues

To explore AMPH1 expression in patients with ovarian cancer, we performed immunohistochemical staining for AMPH1, and calculated the IHC score of AMPH1. Each specimen was scored according to the intensity of the nucleic, cytoplasmic and membrane staining (weak staining = 1, moderate staining = 2 and strong staining = 3) and the proportion of stained cells (0%‐5% = 0, 5%‐25% = 1, 26%‐50% = 2, 51%‐75% = 3 and 76%‐100% = 4). The staining of AMPH1 was remarkably reduced in ovarian cancer tissues compared with normal ovarian tissues (Figure 6A‐C). In addition, the clinicopathologic characteristics of patients and the correlation of AMPH1 demonstrated that AMPH1 was correlated to all the ovarian cancer patients (Figure 6D). We also detected the staining of p‐PI3K and p‐AKT, and the results showed the expression of p‐PI3K and p‐AKT increased in ovarian cancer tissues compared with normal ovarian tissues (Figure 6E).

## 4 DISCUSSION

AMPH1, an abundant protein in nerve terminals, plays a critical role in the recruitment of dynamin to sites of clathrin‐mediated endocytosis.^3^ It is recently reported to be associated with cancer progression, including breast cancer,^10^ and lung cancer.^11^ However, the impact of AMPH1 on ovarian cancer is unclear. Here, this study transfected sh AMPH1 or PCMV‐AMPH overexpression plasmid into ovarian cancer cell lines, Caov‐3 and Skov3 cells, to construct AMPH1 knockdown or AMPH1 overexpression stable cell strains. Our results showed that AMPH1 might function as a tumour suppressor in ovarian cancer via regulating PI3K/AKT signalling pathway.

In detail, we demonstrated that AMPH1 inhibited Caov‐3 and Skov3 cells growth. In addition, AMPH1 promoted caspase‐3 activity, resulting in the increase of cell apoptosis. Ovarian cancer is one of the most aggressive type's gynaecologic cancers in the world characterized by the tendency of metastasizing early.^13^, ^14^ Metastasis is generally connected with poor prognosis. Thus, we detected the connection between AMPH1 and cell migration, and further found that AMH1 prevented cell migration. Xenograft mouse model experiment showed that AMPH1 significantly inhibited ovarian tumour progression. These findings suggest AMPH1 functions as a tumour suppressor, which is consistent with previous studies.^10^, ^11^ However, different from these studies, we not only utilized AMPH1 knockdown cell strain, but also enabled AMPH1 overexpression cell strain to evaluate the association between AMPH1 and ovarian cancer, which is more comprehensive and convincing.

The PI3K/AKT signalling pathway is one of many mechanisms that regulate cell cycle and cell apoptosis, and dysregulation of a component in this pathway leads to cancer.^18^ To further investigate whether PI3K/AKT signalling pathway is involved in the mechanism underlying the anti‐oncogene effects of AMPH1 in ovarian cancer, we evaluated the association between AMPH1 and p‐PI3K or p‐AKT. AMPH1 inhibited the activation of PI3K/AKT signalling pathway in ovarian cancer. This pathway is one of many mechanisms that regulate cell cycle and cell apoptosis, and dysregulation of a component in this pathway leads to cancer.^18^ PI3K mainly phosphorylates lipid‐based phosphatidylinositol secondary messengers upon activation by receptors on the cell surface, and functions as an important regulator of macrophage phagocytosis, and suppression of PI3K inhibits the recruitment of AMPH to the phagocytic cup.^18^, ^19^ AKT binds the PIP prodsucts of PI3K via its pleckstrin homology domain for recruitment to the plasma membrane.^18^ In addition, PI3K/AKT signalling pathway is identified as the primary pathway involved in initiation and regulation of autophagy.^20^ Autophagy is an intracellular lysosomal pathway, involved in protein degradation and organelle degradation.^21^ Interestingly, as another significant form of programmed cell death, autophagy is frequently deregulated in cancer.^22^ Autophagy mediates both cell death promoting and cell death inhibiting activity, which largely depends on cell types and the magnitude of autophagy.^22^ However, excessive autophagy causes cell death.^23^ In this study, AMPH1 inhibited the activation of PI3K/AKT pathway and might eventually induce tumour cell death. This is the first time that AMPH1 is reported to regulate PI3K/AKT signalling pathway.

Finally, we used IHC to detect AMPH1 tumours. IHC score results showed that the staining of AMPH1 was decreased in ovarian cancer tissues compared with normal ovarian tissues, which is consistent with our results in vitro and in vivo that AMPH1 functions as a tumour suppressor in ovarian cancer.

Our study identified AMPH1 as a tumour suppressor in ovarian cancer. The anti‐oncogene effect of AMPH1 may through induce apoptosis via promoting caspase‐3 activity, and suppressing the activation of PI3K/AKT signalling pathway. These findings reveal that AMPH1 may be used as a potential agent for ovarian cancer therapy.

## CONFLICT OF INTEREST

The authors declare no competing financial interests.

## AUTHOR CONTRIBUTIONS

**Yajun Chen:** Conceptualization (equal); Investigation (equal); Writing‐original draft (equal). **Wenjiao Cao:** Investigation (equal). **Lihua Wang:** Conceptualization (equal); Writing‐original draft (equal). **Tianying Zhong:** Conceptualization (equal); Writing‐original draft (equal); Writing‐review & editing (equal).

## Data Availability

All data generated or analysed during this study are included in this article.
